# Association Between Serum Syndecan 1 Levels and Metabolic Syndrome Parameters: A Comparative Cross‐Sectional Study

**DOI:** 10.1002/edm2.70108

**Published:** 2025-10-02

**Authors:** Shima Tavalaie, Saeed Darroudi, Niloofar Shabani, Artemis AzadAra, Ali Mottaghi‐Moghaddam, Mohammad Kalate Rahmani, Maryam Mohammadi‐Bajgiran, Gordon A. Ferns, Maryam Saberi‐Karimian, Majid Ghayour‐Mobarhan

**Affiliations:** ^1^ Metabolic Syndrome Research Center Mashhad University of Medical Sciences Mashhad Iran; ^2^ Student Research Committee, Faculty of Medicine Mashhad University of Medical Sciences Mashhad Iran; ^3^ Department of Epidemiology and Biostatistics, School of Public Health Tehran University of Medical Sciences Tehran Iran; ^4^ Department of Biology, Mashhad Branch Islamic Azad University Mashhad Iran; ^5^ International UNESCO Center for Health‐Related Basic Sciences and Human Nutrition Mashhad University of Medical Sciences Mashhad Iran; ^6^ Brighton and Sussex Medical School Division of Medical Education Brighton UK

**Keywords:** glycocalyx, metabolic syndrome, obesity, proteoglycans, syndecan (SDC)

## Abstract

**Introduction:**

Previous studies have linked Syndecans (SDCs) to hypertension (HTN), BMI and the prevalence of coronary artery disease (CAD). The relationship between SDCs and metabolic syndrome (MetS) has not been explored. This study aimed to investigate the association between serum SDC1 level and MetS.

**Methods:**

This was a comparative cross‐sectional study conducted on the subjects from phase II of the MASHAD study. A total of 81 subjects were divided into three groups: (1) healthy controls (*N* = 26), (2) subjects with MetS and hypertension with serum ALT < 43 U/L (MetS+ HTN+ ALT–), (*N* = 29), and (3) subjects with MetS and hypertension with serum ALT ≥ 43 U/L (MetS+ HTN+ ALT+), (*N* = 26). Serum SDC1 levels were measured using a commercial ELISA kit. Data were analysed using SPSS version 26 and R version 4.0.5 software.

**Results:**

The analysis showed that mean serum SDC1 levels did not significantly differ between healthy and MetS+ groups overall. Among MetS+ subjects, males had significantly higher SDC1 levels than females (17.57 ± 8.48 vs. 12.85 ± 5.59 ng/mL, *p* = 0.018). In the MetS+ HTN+ ALT+ group, males also had higher SDC1 levels compared to females (20.19 ± 10.56 vs. 11.82 ± 5.09 ng/mL, *p* = 0.020), while no significant differences were observed across the MetS groups overall (*p* = 0.474). Additionally, in both the total study sample and the MetS+ HTN+ ALT+ group, SDC1 levels were positively correlated with diastolic blood pressure (DBP) (*r* = 0.256, *p* = 0.021; *r* = 0.463, *p* = 0.017, respectively), with no significant associations found with other metabolic parameters.

**Conclusions:**

These findings suggest that SDC1 levels are higher in males with MetS, particularly those with hypertension and elevated ALT, and are positively associated with DBP in both the total study sample and the MetS+ HTN+ ALT+ group. There were no significant associations with other metabolic parameters. This indicates that SDC1 may serve as a gender‐specific biomarker for vascular risk in MetS, potentially aiding clinicians in identifying high‐risk MetS subjects.

## Graphical abstract

The results revealed that men in the MetS+ HTN+ ALT+ group had significantly higher serum SDC1 levels than women (20.19 ± 10.56 vs. 11.82 ± 5.09 ng/mL, *p* = 0.020). Furthermore, in both the total study sample and the MetS+ HTN+ ALT+ group, SDC1 concentrations were positively correlated with diastolic blood pressure (*r* = 0.256, *p* = 0.021; *r* = 0.463, *p* = 0.017, respectively).

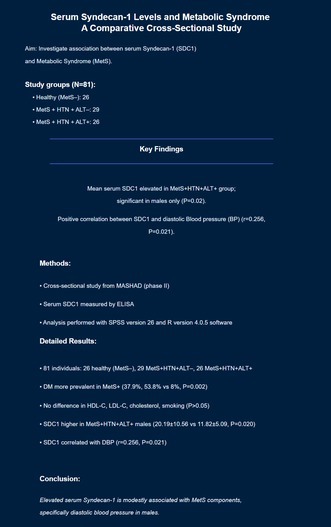

## 1 Introduction

Metabolic syndrome (MetS) is a cluster of interrelated biochemical, clinical and metabolic risk factors, including central obesity, hypertension, glucose intolerance and dyslipidaemia [1]. There is no universally accepted definition for MetS [2]. However, the International Diabetes Federation (IDF), the World Health Organization (WHO) and the National Cholesterol Education Program (NCEP) ATP III state that many risk factors frequently coexist to generate what has become known as MetS [3].

It has been shown that some features of MetS such as dyslipidemia, type 2 diabetes and body composition (specifically fat and muscle mass) have hereditary components. According to estimates, genetic variables account for 70% of fat distribution variation related to MetS and 30%–40% of BMI variance. It is now evident that obesity's underlying genetic aetiology necessitates environmental interaction. Weight gain, a diet rich in saturated fat, tobacco smoking, inactivity and excessive alcohol use are lifestyle variables that raise intra‐abdominal fat and metabolic risk factors [4, 5, 6].

An estimated 20%–25% of adults worldwide are thought to have MetS [7]. The increasing urbanisation in Asia is contributing to a rising prevalence of MetS, with reported rates between 10% and 20%. According to research done in Iran, the prevalence percentage ranges from 28% to 34% [8].

Prospective population studies have shown that the relative risk of CVD events more than doubles in those with MetS [9]. People with MetS have a greater chance of premature death and developing diabetes mellitus or CVD than those without the syndrome. Compared to people without the syndrome, those with MetS have a two to four times greater risk of stroke, a three to four times higher risk of heart attack and twice as high a chance of dying [10].

The main proteoglycans in endothelial Glycocalyx (eGCX) are called syndecans (SDCs), which are type 1 transmembrane proteins that are connected to cell plasma membranes [11]. Several biological processes, such as differentiation, angiogenesis [12], cell adhesion [13] and cell migration [14, 15], are affected by SDCs. However, shedding of syndecan‐1 (SDC1) can be caused by a number of circumstances, including inflammation [16], hyperglycemia [17] and ischemia [18]. SDC1 is expressed by epithelial cells, while syndecan‐2 (SDC2) is expressed by mesenchymal cells. Cartilage and nerve tissue express syndecan‐3 (SDC3) [19], and most tissues express syndecan‐4 (SDC4) [20]. Matrix metalloproteinases (MMPs) are sheddases that perform the shedding of SDCs. MMPs are produced by a variety of cells, such as neutrophils, fibroblasts, endothelial cells, macrophages and lymphocytes. Growth factors control MMPs at both the mRNA and protein levels [21]. MMP‐7 and MMP‐2 are two of the MMPs that contribute to SDC shedding [19].

Previous studies have shown that serum SDC1 levels are higher in people with fatty liver [22]. Another study also demonstrated that serum SDC4 levels are related to metabolic disorders and fatty liver [23]. There have also been prior reports relating SDC4 polymorphisms to the incidence of coronary artery disease (CAD), BMI and hypertension [24].

Since there has not been any research on the relationship between SDCs and MetS up to this point, and because MetS is so common in Iran, we have assessed the relationship between serum levels of SDC1 and MetS in the MASHAD cohort study population.

## 2 Methods

### 2.1 Study Population

This was a comparative cross‐sectional study conducted on the 81 subjects from phase II of the MASHAD cohort study, which started in 2010 and ran until 2020 [25]. The study population comprised a total of 81 subjects, including 26 healthy individuals and 55 patients with MetS whose ALT levels were higher or lower than 43 U/L. Moreover, the study groups were matched for gender in the current investigation. Following the International Diabetic Federation (IDF) criteria 2005 [26], MetS was defined. Participants were divided into the following groups: Group 1: Healthy MetS− (people without MetS with normal blood pressure SBP < 120 mmHg or DBP < 80 mmHg and serum ALT level less than 43 U/L); Group 2: MetS+ and HTN+ and serum ALT < 43 U/L (people with MetS and blood pressure SBP > 120 mmHg or DBP > 80 mmHg); Group 3: MetS+ and HTN+ and serum ALT ≥ 43 U/L (people with MetS and blood pressure SBP > 120 mmHg or DBP > 80 mmHg) [27].

### 2.2 Laboratory and Anthropometric Assessments

A commercial ELISA kit (ZellBio GmbH, Germany; Cat. No. ZB‐13344C‐H9648) was used to measure serum SDC1 levels on stored serum samples that were kept at −80°C. The current comparative cross‐sectional study has used serum samples from the MASHAD cohort study (ID = 951214), which included 26 healthy participants and 55 people with MetS and hypertension.

### 2.3 Ethics

The Ethics Committee at the Mashhad University of Medical Sciences has approved the study protocol, ID = 4020170; IR.MUMS.MEDICAL.REC.1402.268.

### 2.4 Statistical Analysis

Data were analysed using SPSS version 26 and R version 4.0.5 software. Quantitative data were presented as mean ± standard deviation, while qualitative data were summarised using percentages in tables. For statistical analysis and group comparisons, the *t*‐test or its non‐parametric equivalent (Mann–Whitney *U* test) was applied to quantitative variables after assessing the normality of data distribution, while the chi‐squared test was used for categorical variables. Pearson correlation analysis was conducted to examine the linear relationships between serum SDC1 levels and MetS parameters. To evaluate the association between serum SDC1 levels and MetS, both crude (unadjusted) and adjusted multinomial logistic regression models were employed. These association analyses were performed separately for males and females.

## 3 Results

A total of 81 individuals were included in our study: 26 healthy individuals (MetS–), 29 with metabolic syndrome and hypertension but normal ALT levels (MetS+ HTN+ ALT–), and 26 with MetS, hypertension and elevated ALT levels (MetS+ HTN+ ALT+).

Participants in the MetS+ groups (MetS+ HTN+ ALT– and MetS+ HTN+ ALT+) demonstrated significantly higher weight, BMI, waist circumference (WC), fasting blood glucose (FBG), systolic blood pressure (SBP), diastolic blood pressure (DBP) and triglyceride (TG) levels compared to the healthy control group (*p* < 0.001). The prevalence of diabetes mellitus (DM) was significantly higher in the MetS+ groups (MetS+ HTN+ ALT–: 37.9% and MetS+ HTN+ ALT+: 53.8%) compared with the healthy control group (8%, *p* = 0.002). No significant differences were observed in HDL‐C (*p* = 0.106), LDL‐C (*p* = 0.891), total cholesterol (*p* = 0.256) or smoking status (*p* = 0.802). Physical activity levels varied but did not show a significant difference (*p* = 0.105). Table 1 presents baseline characteristics of the population in this study.

**TABLE 1:** Clinical and biochemical characteristics in study population.

Variables		Healthy (*N* = 26)	MetS+ HTN+ (*N* = 29)	MetS+ HTN+ ALT+ (*N* = 26)	*p*
Age (years)		55.92 ± 6.94	60.31 ± 9.71	55.62 ± 8.48	0.076
Weight (kg)		67.60 ± 9.68	77.02 ± 9.70	84.73 ± 15.23	< 0.001
BMI (kg/m^2^)		26.22 ± 3.61	29.67 ± 2.60	32.08 ± 3.92	< 0.001
WC (cm)		85.81 ± 8.27	97.52 ± 5.74	100.58 ± 9.40	< 0.001
FBG (mg/dL)		98 (16)	106 (20.5)	118.5 (39)	< 0.001
Current smoking % (*n*)		7 (26.9)	7 (24.1)	5 (19.2)	0.802
DM % (*n*)		2 (8)	11 (37.9)	14 (53.8)	0.002
SBP (mmHg)		118.54 ± 12.43	146.90 ± 13.49	145 ± 13.84	< 0.001
DBP (mmHg)		70.65 ± 11.66	86.41 ± 11.66	91.42 ± 14.05	< 0.001
HDL‐C (mg/dL)		46.5 (15)	41 (12.5)	37.75 (12.75)	0.106
LDL‐c (mg/dL)		114.65 ± 33.60	113 ± 40.36	118 ± 42.11	0.891
TC (mg/dL)		205.69 ± 47.11	198.69 ± 49.72	220.23 ± 48.69	0.256
TG (mg/dL)		86 (67.75)	148 (116)	172.5 (79.75)	< 0.001
Physical activity level	Sedentary	2 (8)	6 (20.7)	9 (36)	0.105
	Moderately active	6 (24)	9 (31)	6 (24)	
	Vigorously active	9 (36)	8 (27.6)	9 (36)	
	Extremely active	8 (32)	6 (20.7)	1 (4)	

*Note:* Values expressed as mean ± SD for normally distributed data, and median and interquartile range for non‐normally distributed data. Between groups comparisons were assessed by ANOVA for normal distributed data and Kruskal–Wallis for non‐normally distributed data. Median and interquartile range were reported for non‐normally distributed variables, including: FBG, HDL‐C and TG.

Abbreviations: BMI, body mass index; DBP, diastolic blood pressure; DM, diabetes mellitus; FBG, fasting blood glucose; HDL‐C, high‐density lipoprotein cholesterol; LDL‐C, low‐density lipoprotein cholesterol; SBP, systolic blood pressure; TC, Total cholesterol; TG, triglyceride; WC, waist circumference.

Table 2 presents the comparison of mean serum SDC1 levels (ng/mL) across different MetS groups. The mean SDC1 level did not differ significantly between the healthy and MetS+ groups (*p* = 0.983). Within the MetS+ subjects, males exhibited significantly higher mean SDC1 levels (17.57 ± 8.48 ng/mL) than females (12.85 ± 5.59 ng/mL, *p* = 0.018).

**TABLE 2:** Comparison of mean serum SDC1 levels (ng/mL) across different Metabolic syndrome (MetS) groups.

Healthy (*N* = 26)				MetS+				*p*
Total (*N* = 26)	Males (*N* = 11)	Females (*N* = 15)	*p*	Total (*N* = 55)	Males (*N* = 27)	Females (*N* = 28)	*p*	
15.20 ± 4.36	14.92 ± 2.75	15.40 ± 5.34	0.789	15.17 ± 7.48	17.57 ± 8.48	12.85 ± 5.59	0.018	0.983

Healthy (*N* = 26)				MetS+								*p*
				MetS+ HTN+ (*N* = 29)				MetS+ HTN+ ALT+ (*N* = 26)				
Total (*N* = 26)	Males (*N* = 11)	Females (*N* = 15)	*p*	Total (*N* = 29)	Males (*N* = 13)	Females (*N* = 16)	*p*	Total (*N* = 26)	Males (*N* = 14)	Females (*N* = 12)	*p*	
15.20 ± 4.36	14.92 ± 2.75	15.40 ± 5.34	0.789	14.13 ± 5.23	14.78 ± 4.31	13.63 ± 5.97	0.578	16.32 ± 9.35	20.19 ± 10.56	11.82 ± 5.09	0.020	0.474

*Note:* A *p*‐value < 0.05 indicates a significant difference in the mean serum levels of SDC1 between sex groups. Mean ± SD of SDC1 (ng/mL) was reported.

Comparisons among the healthy, MetS+ HTN+ ALT–, and MetS+ HTN+ ALT+ groups showed a slight elevation of serum SDC1 in the MetS+ HTN+ ALT+ group (16.32 ± 9.35 ng/mL) compared with the healthy group (15.20 ± 4.36 ng/mL) and the MetS+ HTN+ ALT– group (14.13 ± 5.23 ng/mL), although this difference was not statistically significant (*p* = 0.474). However, when stratified by sex, male individuals in the MetS+ HTN+ ALT+ group showed significantly higher means of SDC1 levels (20.19 ± 10.56 ng/mL) than females in the same group (11.82 ± 5.09 ng/mL, *p* = 0.020). No significant sex‐related differences were found in SDC1 levels in the other groups (*p* > 0.05). A boxplot illustrating serum SDC1 levels in the MetS groups, stratified by sex, is shown in Figure S1.

**FIGURE 1:**
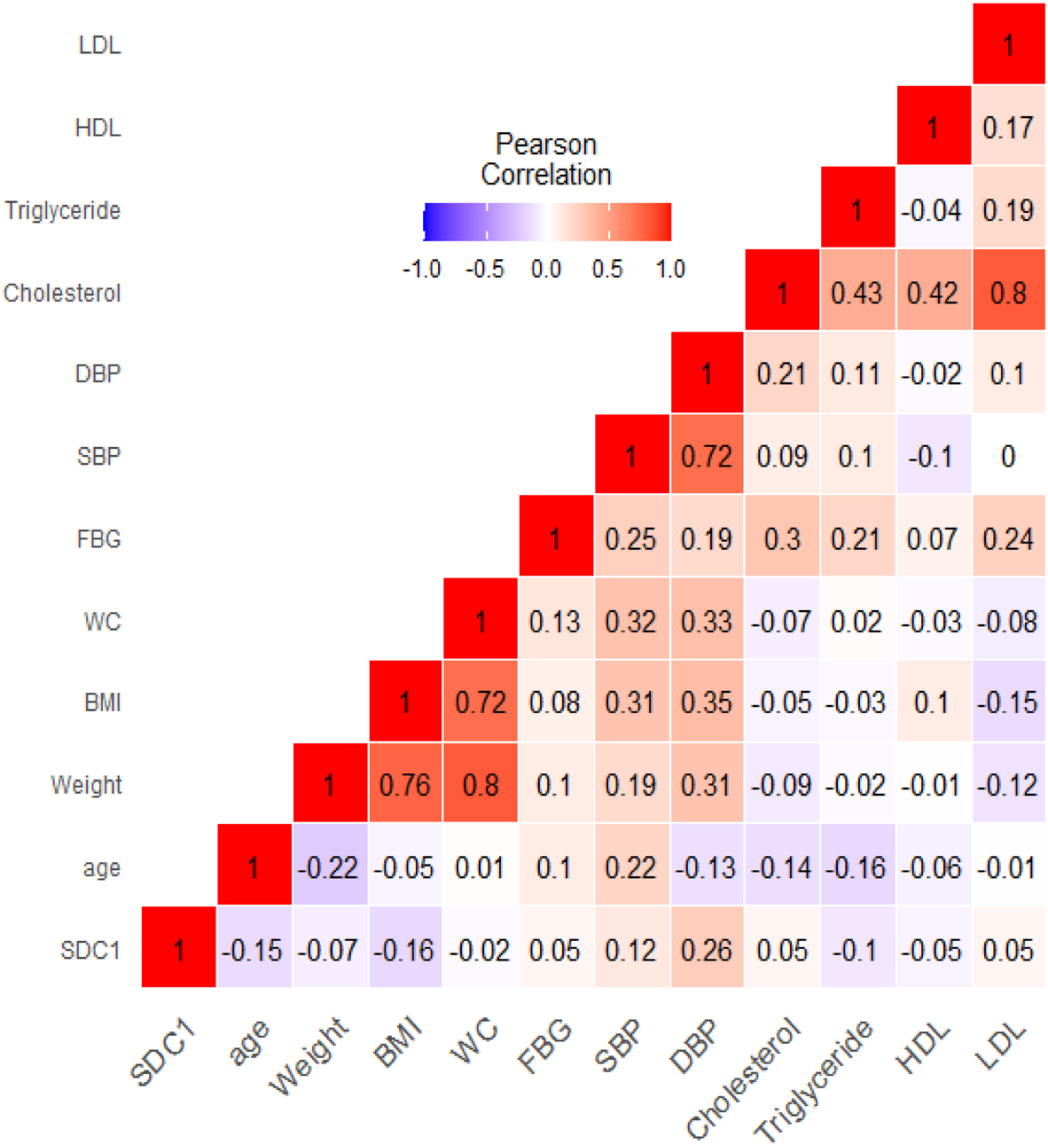
Correlation heatmap of serum syndecan‐1 (SDC1) with Metabolic Syndrome (MetS) parameters in the total study sample. BMI, Body Mass Index; DBP, diastolic blood pressure; FBG, fasting blood glucose; HDL, high density lipoprotein; LDL, low density lipoprotein; SBP, systolic blood pressure; SDC1, syndecan‐1; WC, waist circumference.

Figure 1 illustrates the correlation heatmap of serum SDC1 with MetS parameters. Among the associations, the correlation between SDC1 and DBP was represented by a darker colour compared with other MetS parameters, suggesting a relatively stronger relationship. In the total study sample (81 subjects), correlation analysis revealed a significant positive association between serum SDC1 levels and DBP (*r* = 0.256, *p* = 0.021), indicating that higher SDC1 levels may be linked to increased DBP. No significant correlations were observed between SDC1 and other metabolic markers including BMI (*r* = −0.225, *p* = 0.053), waist circumference (*r* = −0.023, *p* = 0.840), total cholesterol (*r* = 0.051, *p* = 0.651), triglycerides (*r* = −0.098, *p* = 0.384), HDL‐C (*r* = −0.055, *p* = 0.626) or LDL‐C (*r* = 0.049, *p* = 0.662) (see correlation matrix in Table S1). Pearson correlation analysis of serum SDC1 with MetS parameters in each of the different MetS subgroups demonstrates a significant correlation between SDC1 level and DBP in the MetS+ HTN+ ALT+ subgroup (*p* = 0.017) (Table S2).

**FIGURE 2:**
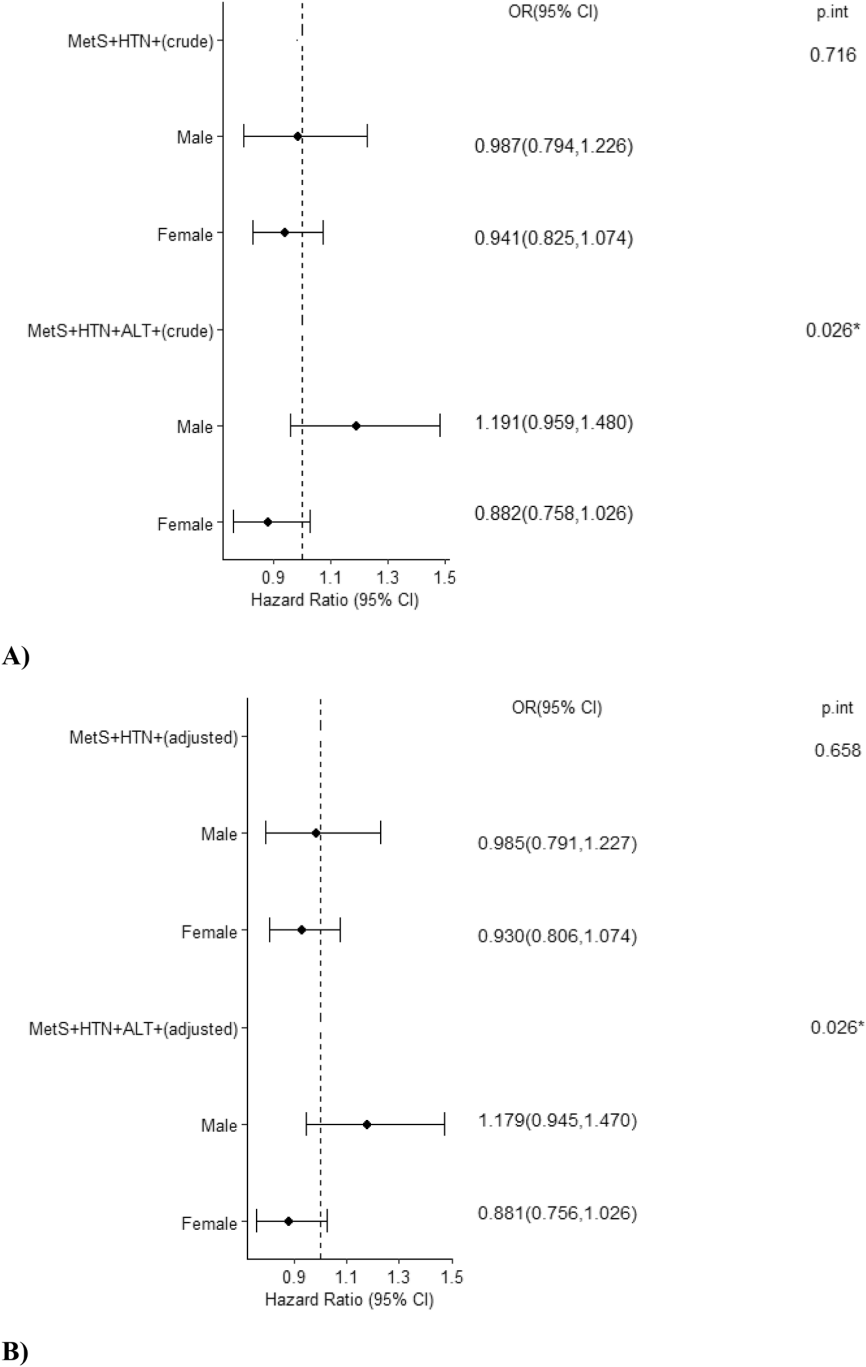
(A) Results of multinomial (crude and adjusted) logistic regression model separated for sex. (B) Model adjusted for age; Reference category = healthy; *p*.int. = *p*‐value interaction (interaction between SDC1 and sex). ALT, alanine amino transferase; HTN, hypertension; MetS, metabolic syndrome; SDC1, syndecan‐1.

Crude (unadjusted) and adjusted multinomial logistic regression models, stratified by sex, were used to assess the predictive role of serum SDC1 levels in MetS classification. Compared with the healthy group, higher SDC1 levels were not significantly associated with greater odds of belonging to either MetS+ subgroup in either the crude or adjusted models. No significant interaction was observed between SDC1 and sex (*p*_int > 0.05) in the MetS+ HTN+ ALT– group. However, this interaction was significant in the MetS+ HTN+ ALT+ group, indicating that the association between SDC1 and MetS classification differed significantly between men and women in this subgroup (Figure 2).

## 4 Discussion

This study is the first focusing on individuals with MetS in the MASHAD cohort to investigate the association between serum SDC1 levels and MetS. While SDC1 has been previously associated with endothelial dysfunction and metabolic dysregulation, our findings provide detailed insights into its specific role within this particular population.

SDC1 plays essential roles in endothelial integrity, inflammation and lipid metabolism that are key pathways involved in MetS. SDC1 modulates leukocyte‐endothelial interactions by establishing chemokine gradients, facilitating leukocyte rolling via selectin binding, and promoting diapedesis through chemokine dimerisation and integrin activation [28]. Its shedding, triggered by inflammatory mediators (e.g., thrombin, hyperglycemia and oxidative stress), releases soluble ectodomains that retain bioactivity, exacerbating endothelial dysfunction and systemic inflammation [29, 30]. In the liver, SDC1 plays a key role in lipoprotein metabolism; it mediates hepatic uptake of chylomicron remnants and VLDL‐C via its heparan sulfate chains, with Sdc1−/− mice exhibiting elevated plasma triglycerides and impaired lipid clearance. Notably, SDC1 shedding in MetS may interfere with this process, as soluble ectodomains compete with cell‐bound SDC1 for lipoprotein binding, potentially contributing to dyslipidaemia [31, 32]. This evidence positions SDC1 as a key mediator in the vicious cycle of endothelial dysfunction, systemic inflammation and metabolic disturbances that characterise MetS.

In a cross‐sectional study of 113 participants, Yilmaz et al. [22] showed that patients with nonalcoholic fatty liver disease (NAFLD) had elevated serum SDC1 levels independently associated with higher HDL‐C. This is consistent with the current study observation of increased SDC1 levels in individuals with MetS, especially those with elevated ALT, and suggests a potential link between SDC1, liver dysfunction and metabolic disorders.

NAFLD, often considered a hepatic manifestation of MetS, further highlights the connection between SDC1 and metabolic dysregulation. Similar to the current study, Cekic et al. [33] reported elevated serum SDC1 levels in patients with active Crohn's disease, associating SDC1 with inflammatory processes that may overlap with metabolic dysfunction. However, their study did not directly examine MetS. The association between SDC1 and vascular risk markers was somewhat confirmed by Yilmaz et al. [22], where an association between SDC1 and HDL‐C was found. This finding is consistent with the present study's finding of a positive correlation between SDC1 and DBP, although other studies have not directly addressed vascular risk. Genetic evidence from Chang et al. [34] study supports the involvement of the SDC family in MetS, showing that the *TT rs2282440* SDC3 genotype is more prevalent among individuals with MetS and its components, including obesity and dyslipidemia. While this study focused on SDC3 rather than SDC1, it highlights the important role of SDCs in metabolic dysregulation.

Contrary to expectations, we found no significant differences in mean SDC1 levels among MetS subgroups (*p* = 0.474). However, a notable sex‐based difference was observed: men in the MetS+ HTN+ ALT+ group had significantly higher SDC1 levels than females (20.19 ± 10.56 vs. 11.82 ± 5.09 ng/mL, *p* = 0.020). In contrast to our findings, another study reported increased serum SDC1 levels in diabetic patients under different insulin regimens compared to controls [35]. This difference may arise from key differences in study populations and metabolic contexts.

A significant sex‐based difference was observed in our study: males in the MetS+ HTN+ ALT+ group had significantly higher SDC1 levels than females. This supports earlier evidence of sexual dimorphism in glycocalyx biology, indicating that hormonal and adipose tissue distribution differences may modulate SDC1 shedding [36].

The only significant correlation was between SDC1 and DBP (*r* = 0.256, *p* = 0.021). This finding aligns with studies indicating SDC1 shedding may contribute to endothelial dysfunction associated with hypertension. Studies have demonstrated that sphingosine‐1‐phosphate (S1P) protects the endothelial glycocalyx by inhibiting SDC1 shedding through suppression of matrix metalloproteinase (MMP) activity. Furthermore, heparanase‐enhanced shedding of SDC1 by myeloma cells has been shown to promote endothelial invasion and angiogenesis [12, 37]. However, we found no significant associations between SDC1 and other metabolic parameters (BMI, WC, lipids). In line with our results, Wang et al. demonstrated that SDC1 has no significant correlation with cholesterol, TG, Lp(a), HDL‐C and LDL‐C [38].

While the sample size in this study is relatively modest, the findings reveal statistically significant and biologically plausible associations between serum SDC1 levels and MetS subgroups, particularly among males with combined hypertension and elevated ALT. The observed sex‐specific disparity in SDC1 levels and its correlation with DBP (*p* = 0.020 and *r* = 0.256, *p* = 0.021, respectively) suggests the study was sufficiently powered to detect these relationships. In biomarker research, exploratory studies often employ smaller sample sizes to identify associations warranting further investigation [39, 40]. Our use of rigorous ELISA methods and well‐defined subgroups enhances the reliability of these findings despite sample size constraints. Meta‐analyses of SDC1 in other contexts (e.g., COVID‐19, trauma) have similarly included smaller studies yet yielded consistent results [41], supporting its utility as a biomarker when stratified appropriately. The sex‐specific differences observed here align with established variations in MetS pathophysiology [42, 43], underscoring SDC1's potential as a biomarker tailored to high‐risk subpopulations.

On the other hand, sex‐related differences in serum SDC1 levels in the MetS+ HTN+ ALT+ subgroup may be explained by the fact that elevated ALT reflects greater hepatic and endothelial stress, conditions in which SDC1 release is more pronounced. Males appear to be more susceptible to ALT‐related endothelial injury, which could account for the higher SDC1 levels in this subgroup [44, 45]. In contrast, in the ALT‐ subgroup, where hepatic stress was less pronounced, no significant sex difference was detected. However, in the multinomial logistic regression analysis, the interaction between sex and SDC1 across MetS classifications was not significant. This suggests that while subgroup analyses point to a possible sex‐specific pattern, the effect is not robust when adjusted for other metabolic factors, likely due to the limited sample size. Larger studies are needed to clarify the role of sex in the relationship between SDC1 and metabolic abnormalities.

To our knowledge, no previous studies have examined sex‐specific differences in serum SDC1 levels in MetS or related settings. This highlights the novelty of our finding in the MetS+ HTN+ ALT+ subgroup and underscores the need for future research to validate sex‐specific mechanisms in endothelial and hepatic stress responses. Moreover, this study has limitations, including its cross‐sectional design (precluding causal inferences) and moderate sample size, which may affect generalisability. Future studies should examine SDC1's temporal relationship with MetS progression, while mechanistic work could explore the sex‐specific differences we observed. Integrating genetic and multi‐omics data may further clarify SDC1's role in metabolic dysfunction.

In conclusion, while larger studies are needed for validation, our findings provide preliminary evidence supporting SDC1's role in MetS, particularly in males with hypertension and elevated ALT. These results justify further investigation of SDC1 as a gender‐specific biomarker and potential therapeutic target in metabolic and vascular risk stratification.

## 5 Conclusion

These findings collectively highlight a significant association between SDC1 and MetS, particularly in males with combined hypertension and elevated ALT. The sex‐specific disparity and SDC1‐DBP correlation underscore SDC1's potential as a gender‐stratified biomarker for vascular risk in MetS. Future studies should investigate the role and therapeutic value of SDC1 in high‐risk MetS subgroups.

## Author Contributions

Study Design, Shima Tavalaie, Saeed Darroudi, Maryam Saberi‐Karimian, Majid Ghayour‐Mobarhan. Data Collection, Ali Mottaghi‐Moghaddam, Mohammad Kalate Rahmani, Maryam Mohammadi‐Bajgiran. Statistical Analysis, Niloofar Shabani. Data Interpretation, Shima Tavalaie, Saeed Darroudi, Maryam Saberi‐Karimian, Majid Ghayour‐Mobarhan, Niloofar Shabani. Manuscript Preparation, Shima Tavalaie, Saeed Darroudi, Artemis AzadAra, Maryam Saberi‐Karimian, Majid Ghayour‐Mobarhan, Gordon A. Ferns. Literature Search, Shima Tavalaie, Saeed Darroudi, Maryam Saberi‐Karimian, Majid Ghayour‐Mobarhan. Funds Collection, Maryam Saberi‐Karimian.

## Conflicts of Interest

The authors declare no conflicts of interest.

## Supporting information


**Appendix S1:** edm270108‐sup‐0001‐AppendixS1.docx.

## Data Availability

The data that support the findings of this study are available from the corresponding author upon reasonable request.
