# Supplementary material for: Association Between Serum Syndecan 1 Levels and Metabolic Syndrome Parameters: A Comparative Cross‐Sectional Study
Source: Endocrinol Diabetes Metab. 2025 Oct 2;8(6):e70108. doi: 10.1002/edm2.70108 (PMC12491147; doi:10.1002/edm2.70108)

**Appendix S1**

| **Table S1:** Correlations between serum SDC1 and MetS parameters. | | | | | | | | | | | | | |
| --- | --- | --- | --- | --- | --- | --- | --- | --- | --- | --- | --- | --- | --- |
|  | | SDC1 | Age | Weight | BMI | WC | FBG | SBP | DBP | Total cholesterol | Triglyceride | HDL-C | LDL-C |
| SDC1 | Pearson Correlation | 1 | -.146 | -.066 | -.225 | -.023 | .049 | .119 | .256^*^ | .051 | -.098 | -.055 | .049 |
|  | Sig. (2-tailed) |  | .195 | .558 | .053 | .840 | .667 | .291 | .021 | .651 | .384 | .626 | .662 |
|  | N | 81 | 81 | 81 | 75 | 81 | 81 | 81 | 81 | 81 | 81 | 81 | 81 |
| Age | Pearson Correlation | -.146 | 1 | -.223^*^ | -.051 | .015 | .099 | .218 | -.127 | -.136 | -.161 | -.062 | -.010 |
|  | Sig. (2-tailed) | .195 |  | .045 | .666 | .897 | .377 | .051 | .257 | .225 | .151 | .585 | .930 |
|  | N | 81 | 81 | 81 | 75 | 81 | 81 | 81 | 81 | 81 | 81 | 81 | 81 |
| Weight | Pearson Correlation | -.066 | -.223^*^ | 1 | .773^**^ | .803^**^ | .103 | .185 | .306^**^ | -.086 | -.021 | -.014 | -.116 |
|  | Sig. (2-tailed) | .558 | .045 |  | .000 | .000 | .359 | .098 | .005 | .446 | .849 | .900 | .301 |
|  | N | 81 | 81 | 81 | 75 | 81 | 81 | 81 | 81 | 81 | 81 | 81 | 81 |
| BMIBodyMassIndex | Pearson Correlation | -.225 | -.051 | .773^**^ | 1 | .739^**^ | .082 | .320^**^ | .378^**^ | -.056 | -.026 | .114 | -.165 |
|  | Sig. (2-tailed) | .053 | .666 | .000 |  | .000 | .485 | .005 | .001 | .631 | .827 | .332 | .156 |
|  | N | 75 | 75 | 75 | 75 | 75 | 75 | 75 | 75 | 75 | 75 | 75 | 75 |
| WC | Pearson Correlation | -.023 | .015 | .803^**^ | .739^**^ | 1 | .129 | .325^**^ | .329^**^ | -.070 | .018 | -.027 | -.079 |
|  | Sig. (2-tailed) | .840 | .897 | .000 | .000 |  | .251 | .003 | .003 | .533 | .874 | .809 | .485 |
|  | N | 81 | 81 | 81 | 75 | 81 | 81 | 81 | 81 | 81 | 81 | 81 | 81 |
| FBG | Pearson Correlation | .049 | .099 | .103 | .082 | .129 | 1 | .246^*^ | .191 | .299^**^ | .209 | .067 | .245^*^ |
|  | Sig. (2-tailed) | .667 | .377 | .359 | .485 | .251 |  | .027 | .088 | .007 | .061 | .555 | .028 |
|  | N | 81 | 81 | 81 | 75 | 81 | 81 | 81 | 81 | 81 | 81 | 81 | 81 |
| SBP | Pearson Correlation | .119 | .218 | .185 | .320^**^ | .325^**^ | .246^*^ | 1 | .724^**^ | .091 | .099 | -.102 | .000 |
|  | Sig. (2-tailed) | .291 | .051 | .098 | .005 | .003 | .027 |  | .000 | .417 | .381 | .365 | .999 |
|  | N | 81 | 81 | 81 | 75 | 81 | 81 | 81 | 81 | 81 | 81 | 81 | 81 |
| DBP | Pearson Correlation | .256^*^ | -.127 | .306^**^ | .378^**^ | .329^**^ | .191 | .724^**^ | 1 | .214 | .108 | -.025 | .097 |
|  | Sig. (2-tailed) | .021 | .257 | .005 | .001 | .003 | .088 | .000 |  | .056 | .337 | .826 | .389 |
|  | N | 81 | 81 | 81 | 75 | 81 | 81 | 81 | 81 | 81 | 81 | 81 | 81 |
| Total cholesterol | Pearson Correlation | .051 | -.136 | -.086 | -.056 | -.070 | .299^**^ | .091 | .214 | 1 | .431^**^ | .422^**^ | .802^**^ |
|  | Sig. (2-tailed) | .651 | .225 | .446 | .631 | .533 | .007 | .417 | .056 |  | .000 | .000 | .000 |
|  | N | 81 | 81 | 81 | 75 | 81 | 81 | 81 | 81 | 81 | 81 | 81 | 81 |
| Triglyceride | Pearson Correlation | -.098 | -.161 | -.021 | -.026 | .018 | .209 | .099 | .108 | .431^**^ | 1 | -.040 | .194 |
|  | Sig. (2-tailed) | .384 | .151 | .849 | .827 | .874 | .061 | .381 | .337 | .000 |  | .723 | .083 |
|  | N | 81 | 81 | 81 | 75 | 81 | 81 | 81 | 81 | 81 | 81 | 81 | 81 |
| HDL-C | Pearson Correlation | -.055 | -.062 | -.014 | .114 | -.027 | .067 | -.102 | -.025 | .422^**^ | -.040 | 1 | .172 |
|  | Sig. (2-tailed) | .626 | .585 | .900 | .332 | .809 | .555 | .365 | .826 | .000 | .723 |  | .125 |
|  | N | 81 | 81 | 81 | 75 | 81 | 81 | 81 | 81 | 81 | 81 | 81 | 81 |
| LDL-C | Pearson Correlation | .049 | -.010 | -.116 | -.165 | -.079 | .245^*^ | .000 | .097 | .802^**^ | .194 | .172 | 1 |
|  | Sig. (2-tailed) | .662 | .930 | .301 | .156 | .485 | .028 | .999 | .389 | .000 | .083 | .125 |  |
|  | N | 81 | 81 | 81 | 75 | 81 | 81 | 81 | 81 | 81 | 81 | 81 | 81 |

**Table S2.** Pearson correlation analysis of serum Syndecan-1(SDC1) with Metabolic Syndrome (MetS) parameters in different MetS groups

|  | **Healthy (N=26)** | | **MetS+HTN+ (N=29)** | | **MetS+HTN+ALT+ (N=26)** | |
| --- | --- | --- | --- | --- | --- | --- |
|  | **Pearson Correlation** | **P-value** | **Pearson Correlation** | **P-value** | **Pearson Correlation** | **P-value** |
| **Age** | -0.028 | 0.891 | -0.039 | 0.841 | -0.226 | 0.266 |
| **Weight** | -0.326 | 0.105 | -0.207 | 0.282 | -0.016 | 0.936 |
| **BMI** | -0.348 | 0.088 | -0.237 | 0.244 | -0.142 | 0.508 |
| **WC** | -0.155 | 0.451 | -0.104 | 0.592 | 0.019 | 0.927 |
| **FBG** | -0.107 | 0.602 | -0.005 | 0.979 | 0.115 | 0.574 |
| **SBP** | 0.166 | 0.419 | 0.190 | 0.323 | 0.195 | 0.341 |
| **DBP** | -0.055 | 0.788 | 0.265 | 0.165 | 0.463 | **0.017*** |
| **Total cholesterol** | -0.094 | 0.650 | 0.118 | 0.543 | 0.026 | 0.901 |
| **Triglyceride** | -0.093 | 0.650 | -0.242 | 0.205 | -0.147 | 0.475 |
| **HDL-C** | -0.174 | 0.395 | 0.022 | 0.908 | -0.096 | 0.641 |
| **LDL-C** | 0.034 | 0.870 | 0.099 | 0.611 | 0.015 | 0.943 |

**Figure S1:** Boxplot of SDC1 (ng/mL) in Different Metabolic Syndrome Groups, Stratified by Sex.

Male


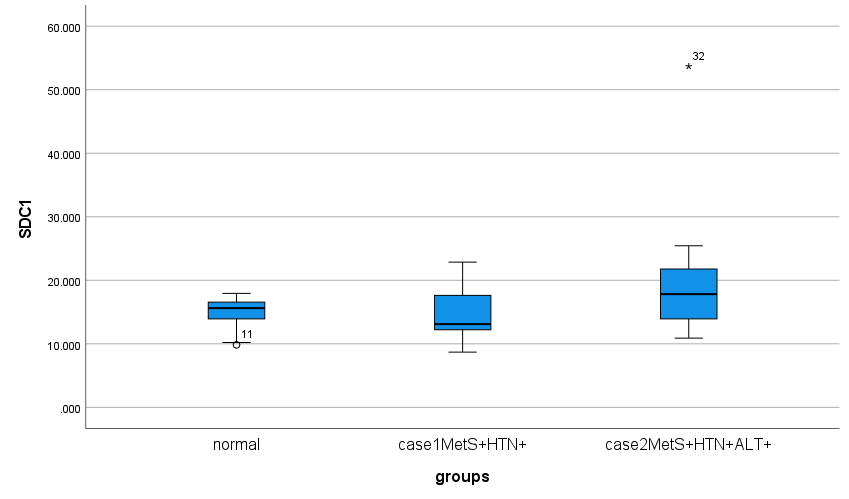


Female


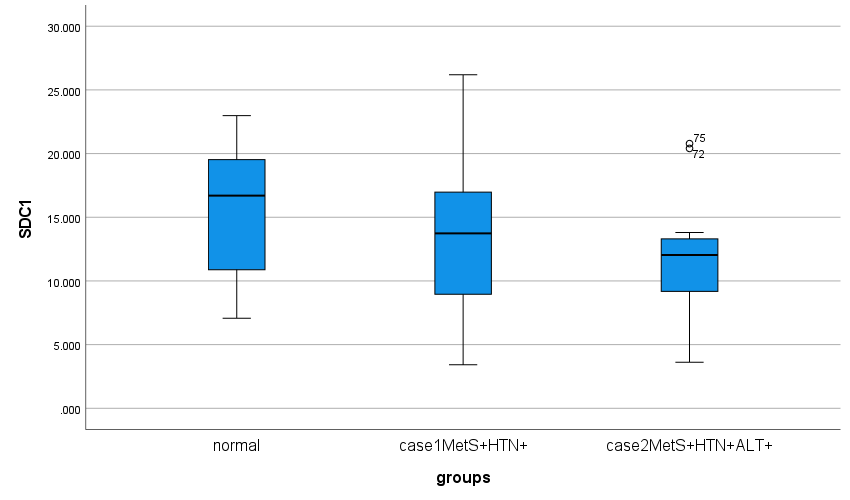

Supplement: Supplementary file 1 — Appendix S1: edm270108‐sup‐0001‐AppendixS1.docx. [file EDM2-8-e70108-s001.docx]
